# Neuropsychiatric Manifestations of Tuberous Sclerosis in a young adult male in a Psychiatry Hospital in Botswana: a case report

**DOI:** 10.1192/j.eurpsy.2024.1027

**Published:** 2024-08-27

**Authors:** W. T. Chabalala, K. Bojosi, T. Maphane, A. Olashore

**Affiliations:** ^1^Department of Psychiatry, University of Botswana; ^2^Department of Psychiatry, Sbrana Psychiatry Hospital, Gaborone, Botswana

## Abstract

**Introduction:**

Tuberous sclerosis complex (TSC) is a disorder that affects multiple systems and was first described in 1880. Its symptoms include seizures, intellectual disability, and adenoma sebaceum. TSC is caused by mutations in the TSC1 and TSC2 genes and is inherited in an autosomal dominant manner.

**Objectives:**

This report highlights a case of a patient with an unusual psychological presentation evaluated in a psychiatric hospital.

**Methods:**

The patient presented with psychotic features and abnormal behavior. A physical examination showed neurocutaneous lesions. After assessment a diagnosis of Tuberous sclerosis complex was confirmed through MRI Brain and genetic testing. Some of his relatives also showed similar neuropsychiatric symptoms.

**Results:**

Tuberous sclerosis complex is diagnosed based on TSC Clinical Consensus Group guidelines of 2012. Our patient fulfilled 4 of the major criteria and genetic testing also yielded a pathogenic variant. A TAND checklist (TSC-Associated Neuropsychiatric Disorders) is used to guide clinicians on areas to prioritize when managing TSC patients.

**Image:**

**Figure:**
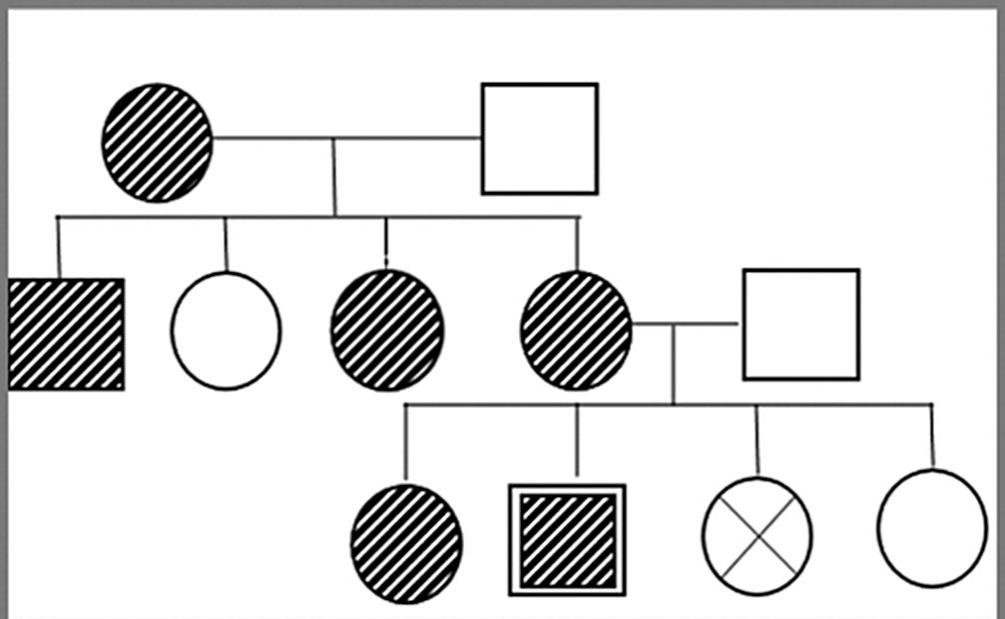

**Image 2:**

**Figure:**
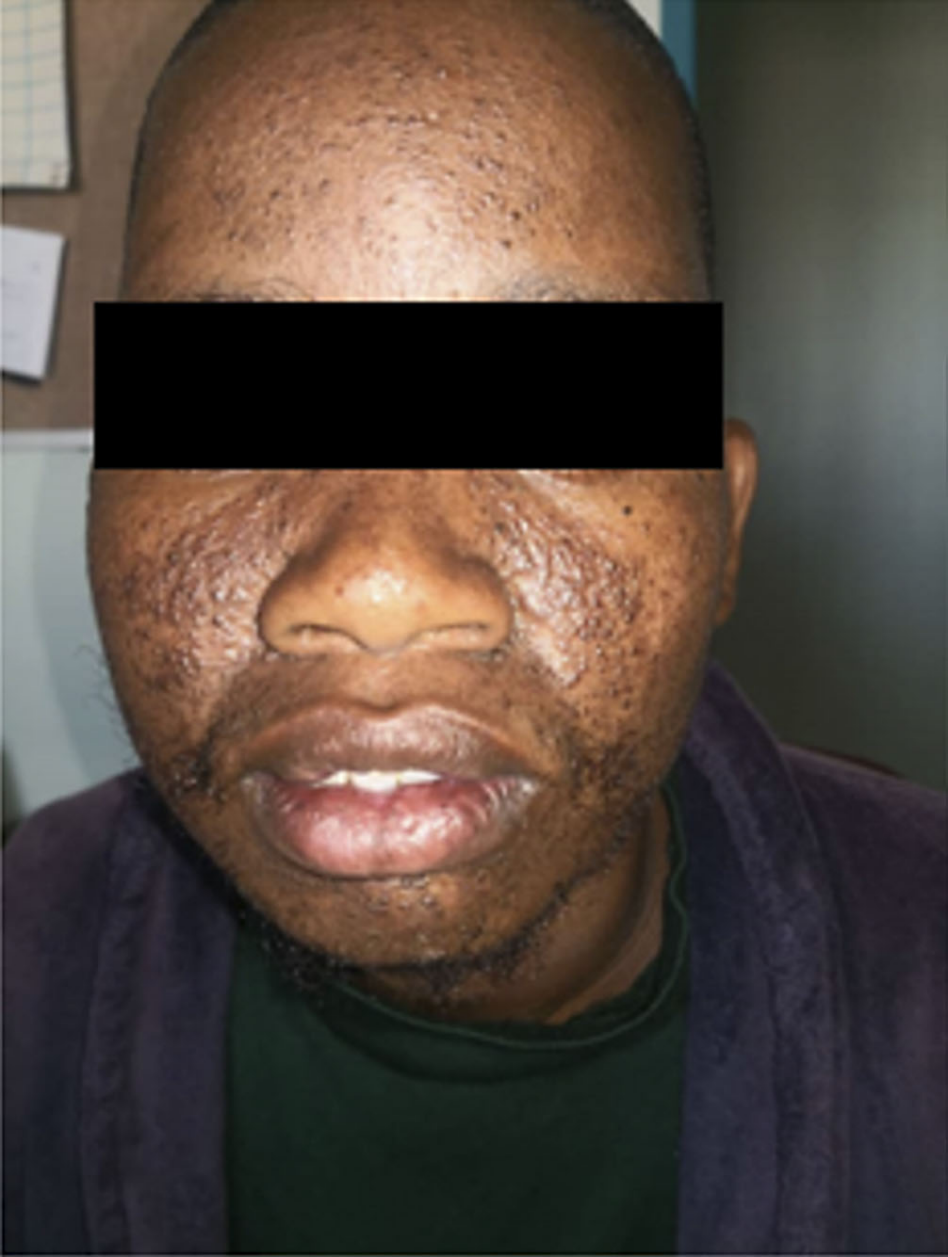

**Image 3:**

**Figure:**
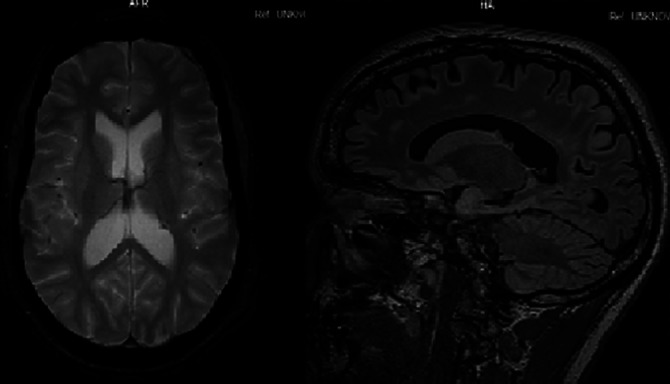

**Conclusions:**

Given that psychiatry may be the first contact for TSC patients, especially in low-resource settings. Patients referred to psychiatry, therefore, need to be thoroughly examined to exclude neuropsychiatric disorders, and a multidisciplinary team approach is vital in investigating and managing these cases.

**Disclosure of Interest:**

None Declared

